# Features of Natural Succession of Ex-Arable Forest Steppe Grassland (from Western Romania) under the Influence of Climate

**DOI:** 10.3390/plants12061204

**Published:** 2023-03-07

**Authors:** Veronica Sărățeanu, Otilia Cotuna, Mirela Paraschivu, Luminița L. Cojocariu, Nicolae Marinel Horablaga, Dorin Rechițean, Vlad Dragoslav Mircov, Călin Sălceanu, Alina Andreea Urlică, Loredana Copăcean

**Affiliations:** 1 Agriculture Faculty, University of Life Sciences “King Michael I” from Timisoara Calea Aradului Street, No. 119, 300645 Timișoara, Romania; 2 Agricultural Research and Development Station Lovrin, Street Principală, No. 200, 307250 Lovrin, Romania; 3 Faculty of Agronomy, Department of Agriculture and Forestry Technologies, University of Craiova, A.I. Cuza Street, No. 13, 200585 Craiova, Romania

**Keywords:** ex-arable grassland, forest steppe, natural succession, climate, temperature, rainfalls, floristic composition, biodiversity, pastoral value

## Abstract

Important land surfaces from hill and mountain areas from the northern hemisphere formerly used for cropping were abandoned. Often, the abandoned land evolved by natural succession to grassland, shrubland or even to forest. The main goal of this paper is to bring new datasets necessary for the understanding of the evolution of ex-arable grassland vegetation from the forest steppe area into relationship with climate. The researches were performed in the locality of Grădinari (Caraş-Severin County, Western Romania) on an ex-arable plot abandoned since 1995. The vegetation data were collected for 19 years (time interval 2003–2021). The analyzed vegetation features were floristic composition, biodiversity and pastoral value. The climate data considered were air temperature and rainfall amount. The vegetation and climate data were correlated statistically, with a view to highlighting the potential impact of the temperature and rainfalls during the evolution of succession process on the grassland’s floristic composition, biodiversity and pastoral value. The pressure of the increased temperatures on the natural restoration process of the biodiversity and pastoral value of ex-arable forest steppe grassland could, at least partially, be mitigated by random grazing and mulching works.

## 1. Introduction

The reclamation of every abandoned arable land plot has great importance, because the intensity of arable land loss is very dynamic [1]. Some assumptions claiming that at worldwide level in about the last 40 years already lost about a third from the total arable land, mainly due to soil erosion and pollution [1,2]. Other reasons for the abandonment of the arable land in the northern hemisphere are the location of the fields in hill and mountain areas, fragmentation and transition of the ownership from public to private (e.g., in the former communist countries) [3,4,5], population migration from rural to urban areas [6,7,8], etc. Thus, the restoration of ex-arable land as successional secondary grassland [4,9,10,11,12,13,14,15] is one of the ways of successful recovery of the economical, ecological and even social functions [3,10,13,15,16,17,18,19] of these land surfaces. The great importance of the ex-arable land reclamation by turning it in grassland consists in numerous advantages. The restored ex-arable grassland provides conservation of biodiversity at the landscape level [16,20,21], is a cheap forage/biomass resource [3,17,18,22], provides ecological services [6,7,12,16,19,23], enhances carbon sequestration in soil [1,24,25,26,27,28], is a genetic pool of biodiversity [13,18] and provides habitat and refuge for wild fauna [6,16,29,30,31], etc.

According to Schmid et al. [16], a classification of ex-arable successional grasslands can be constructed considering the time past from the abandonment, namely, early successional grassland (5–14 and 15–49 years), mid-successional grassland (50–278 years) and late successional grassland (≥280 years).

The research results from the literature regarding grassland restoration from ex-arable land are various due to the implication of numerous approaches. There are dominate random solutions for the application of different techniques for the restoration of the abandoned arable land, such as the input of seeds from grasslands from neighboring area [13], or the input of commercial seeds [15,32,33], the application of top soil from late successional grasslands [27], the application of nitrogen and litter [34], the introduction of the large herbivores [35], etc. It is well known that climate [36,37] and soil [38], followed by management intensity, are the main drivers for the existence of a certain type of grassland [39] and are directly implied in the succession and fluctuation of the grassland vegetation [3,4,11,14,40,41,42]. In the conditions of climate change intensification from nowadays, the restoration of the abandoned arable land can be one of the most important and effective actions for the mitigation of the dramatic consequences of it at worldwide level [43].

In Eastern Europe, forest steppe is an important mosaic-like forest-grassland habitat from lowland areas enabled primarily by the semi-arid and semi-humid climate [31]. The ecological conditions provided by the forest steppe were proper for the development of the agriculture from the Neolithic era to the modern days, when often the forest steppe became agricultural land cultivated intensively [44]. Forest steppe has high specific biodiversity, numerous endemic and rare species and ancestral genotypes of the cultivated plants [31,45]. These are one of the most fragmented and endangered habitats from Eastern Europe [16,31] and from the entire northern hemisphere [45,46].

In this work, we have investigated aspects regarding the influence of climate on some features of a successional ex-arable forest steppe grassland in conditions of natural recovery of the vegetation sward. The hypothesis from the background of the present research presumes the climate impact on some functional agro-ecological features of an ex-arable grassland as floristic composition, biodiversity and pastoral value in a time frame of 19 years. The objectives of the research were the following: (i) characterization of the vegetation sward (floristic composition, biodiversity and pastoral value); (ii) general characterization of the climate; and (iii) analysis of the climate impact on the successional vegetation considered features.

The investigation refers to the analysis of the impact of temperatures and rainfalls on grassland vegetation features, for the identification of some key outputs implied in the development of the forest steppe grassland from abandoned arable land at an early successional stage of natural restoration.

Thus, in the context of the actual climate evolution, it seems possible to have a good natural recovery of the biodiversity and pastoral value of ex-arable forest steppe grassland vegetation in the conditions of applying a minimal management as random grazing and mulching works; some results that support this hypothesis are presented in this work.

## 2. Results

### 2.1. Vegetation Dynamics

At the beginning of the research, in the year 2003, the abandoned arable land from Grădinari was in the 90th year of abandonment. At that moment, the vegetation was characterized by a high rate of field weed species, the dominant one being *Bromus hordeaceus* (CS% = 29.22). At the same time, the relatively high contribution of the perennial grassland species *Lolium perenne* (CS% 1 = 8.18), *Calamagrostis epigeios* and *Cynodon dactylon* (CS% = 5.19) was already indicating the ongoing succession from abandoned arable field to successional grassland. *Bromus hordeaceus* was the dominant grass species until the year 2006 when its place was categorically taken by *Festuca valesiaca* with CS% = 34.66. Since 2007, *F. valesiaca* has been accompanied by *Agrostis tenuis*, both dominating the vegetation cover in all the following experimental years (see Table A1, Appendix A). Thus, the forest steppe grassland species started to establish itself massively from the year 2005 in the experimental area. Some of the late successional forest steppe grassland species found in the first years of research were *Festuca valesiaca*, *Filipendula vulgaris*, *Fragaria viridis*, *Vicia cracca*, *Poa pratensis*, *Euphorbia cyparissias*, etc. Later, the number of forest steppe grassland species increased more and more with variations from year to year, there being found, e.g., *Stachys* (*Betonica*) *officinallis* (2009), *Lathyrus pratensis* (2010), *Rosa gallica* (2011), *Botriochloa ischaemum* (2012), *Stipa capillata* (2015), *Salvia pratensis* (2017), *Brachipodium pinnatum* (2020), etc. The taxa inventory is presented in Table A1 (see Appendix A), the species being grouped per years and by grassland functional groups (grasses, legumes and forbs).

### 2.2. Climate

**Figure 1:**
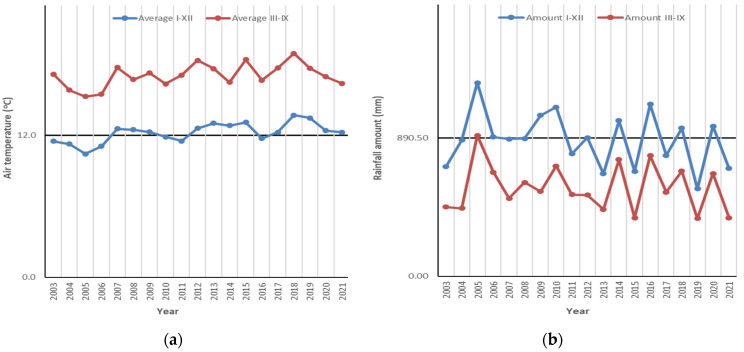
Climate data from the nearest meteorological station (Oravița, Caraș-Severin County) during 2003–2021 time interval (average value during the experiment displayed): (**a**) air temperature (°C); (**b**) rainfall amount (mm) (Source data: Oravița Meteorological Station).

The climate dynamics during the research period were characterized by exceptional values compared with the multiannual average values. The most dramatic evolutions were noticed in the case of air temperatures (Figure 1a). The exceptional temperatures were above the average value from the experimental period more frequently in the last decade, namely in 9 years, and were above the 1955–2012 multiannual average value (11.21 °C) in almost all the years from the studied interval, except 2005 (10.43 °C) and 2006 (10.08 °C), those being the coolest years from the considered timeframe 2003–2021. The annual average temperatures overpassed 13 °C in several years, respectively, in 2013 (13.01 °C), 2015 (13.08 °C), 2018 (13.40 °C) and 2019 (13.45 °C), the hottest years from the analyzed time interval. Considering the data from the vegetation season (months III–IX), the coolest period was measured in 2005 and the hottest in 2018. The most exceptional monthly average air temperature was 28.3 °C in June 2018, with 9.2 °C above the multiannual average value of this month.

The situation regarding the rainfalls was not so dramatic as it was for the temperatures (Figure 1b). Thus, the rainfall amounts were even greater, with 9 years overpassing the 1955–2015 multiannual rainfall average amount of 890.5 mm. The driest year during the research was 2019 (562.5 mm) and the wettest was 2005 (1244.8 mm).

The wettest months registered during the research were July 2011 (238.2 mm) and July 2020 (234.2 mm), while the multiannual average value for July is 101.4 mm. The driest months were July 2015 (1 mm) and October 2013 (4 mm).

**Figure 2:**
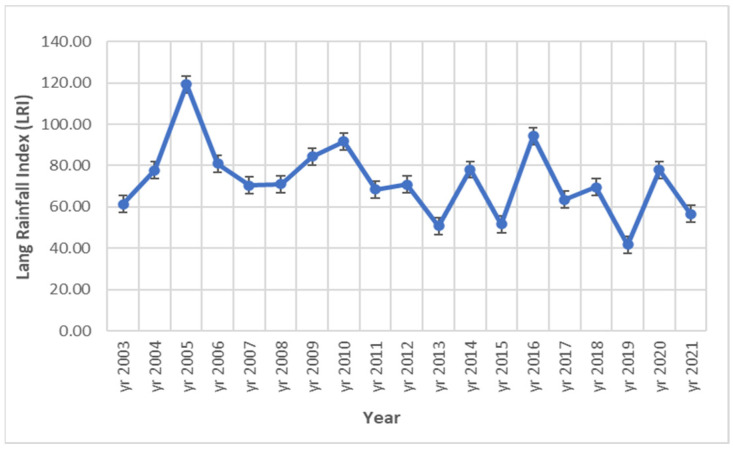
Dynamics of Lang Rainfall Index (R) during 2003–2021 time interval in ex-arable grassland from Grădinari (standard error displayed).

Thus, in general the values of air temperature had an increasing trend. In the case of rainfalls, there was a decrease near to the end of the studied time interval, but in the same time they were characterized by an irregular accentuated variation from one year to the next. In this context, it was considered that the conection between the analyzed climate parameters can be expressed with the Lang Rainfall Index (R). For the experimental site, the R values obtained during 2003–2021 show a high variation, as they fell between 41.82–119.31 (Figure 2). According to the classification of the R values as characteristic climate types, the lowest values falling in the interval 40–60 were obtained toward the end of the analyzed interval (years 2013, 2015, 2019 and 2021), these values corresponding to the semiarid climate. In 74% from the analyzed cases, covering 14 years from the study interval, the R values were framed between 60–100, corresponding to the warm temperate climate. Values of R greater than 100, characteristic of the humid temperate climate, were obtained in a single case, namely in the year 2005.

### 2.3. Biodiversity Dynamics and Relationship with Air Temperature and Rainfall

**Figure 3:**
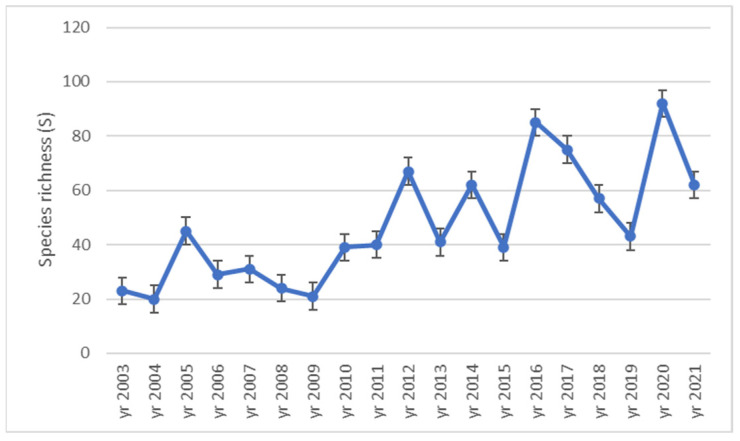
Dynamics of species richness (S) during 2003–2021 time interval in ex-arable grassland from Grădinari (standard error displayed).

Biodiversity was analyzed during the experiment by the species richness (S), the Shannon index (H′) and the Simpson index (D). The species richness had a general increasing trend during the research (Figure 3). The lowest species richness was registered in 2004 (20 species) and the highest in 2020 (92 species). The evolution of the species richness from the ex-arable grassland from Grădinari proves that the number of taxa increases throughout the research period.

**Figure 4:**
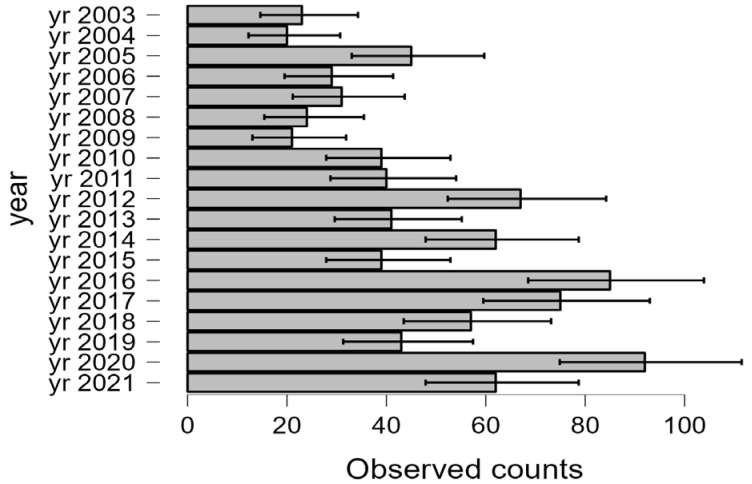
Species richness (S) multinomial test plot for 2003–2021 time interval in ex-arable grassland from Grădinari.

The species richness corresponds to the expected yearly distribution according to the multinomial test outputs (*p* < 0.001). The number of taxa appeared to be influenced by the year (Figure 4). Climate is one of the independent features of interest for our investigation that can be characterized yearly in relationship with the vegetation features and presented in the following chapters of the work.

**Figure 5:**
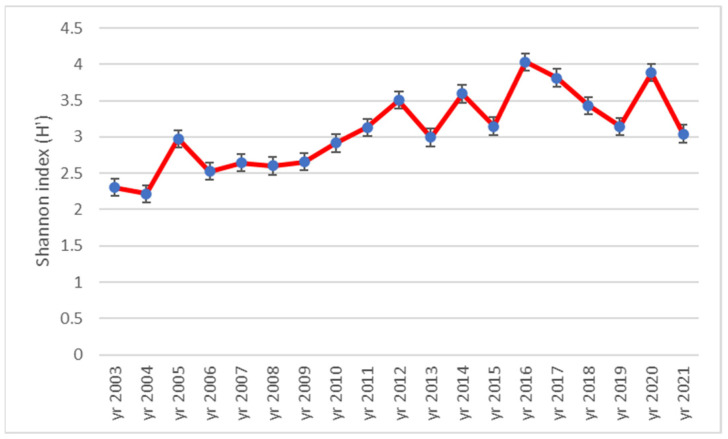
Dynamics of Shannon index (H′) during 2003–2021 time interval in ex-arable grassland from Grădinari (standard error displayed).

Figure 5 presents the evolution of the Shannon index (H′) during the research time interval. The trend highlighted is similar as in the case of the species richness. The lowest H′ value was 2.22 in the year 2004 and the highest was 4.03 in 2016. In general, the Shannon indexes obtained during the research proved the increase in the biodiversity in a great measure from low to high and even with respect to very high biodiversity (values of H′ above 3.5).

**Figure 6:**
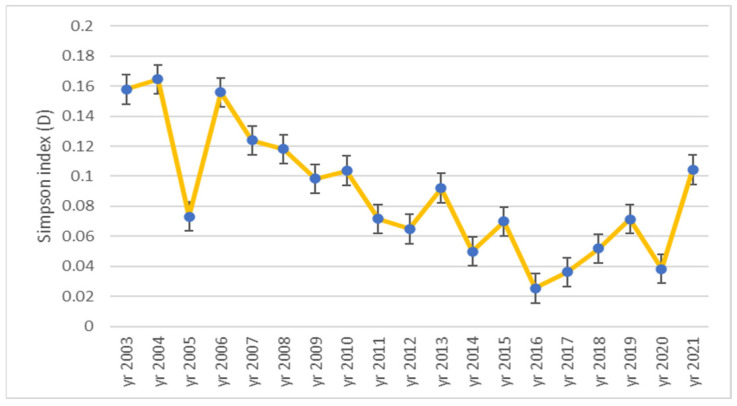
Dynamics of Simpson index (D) during 2003–2021 time interval in ex-arable grassland from Grădinari (standard error displayed).

The Simpson index (D) has a little different pattern as can be noticed in Figure 6, but the meaning is similar, with the greatest biodiversity being in 2016 (D = 0.03) and the lowest in 2003, 2004 and 2006 (D = 0.16), respectively. Simpson’s dominance index is inverse proportional to the Shannon index, the results obtained being near 0 and characteristic for very high diversity (D = 0 represents infinite diversity; D = 1 represents no diversity).

The results regarding the correlation coefficients between the temperatures and biodiversity indexes are presented in Table 1. It was noticed that the air temperature from February (TA II) was positively correlated with the Shannon index (*r* = 0.479 *). The other correlation identified was a negative one and showed that the increase in the air temperature in September determined the decrease in the Simpson index (*r* = −0.498 *).

After the analysis referring to the existence of the correlation between the rainfall amount and the considered biodiversity indexes (Table 2), no statistically significant correlation was identified.

### 2.4. Florisic Composition Dynamics and Relationship with Air Temperature and Rainfall

Floristic composition analysis of the successional ex-arable forest steppe grassland grouped the taxa in three functional groups, namely grasses, legumes and forbs. They were characterized according to species number (no.) and specific contribution rate (CS%).

**Figure 7:**
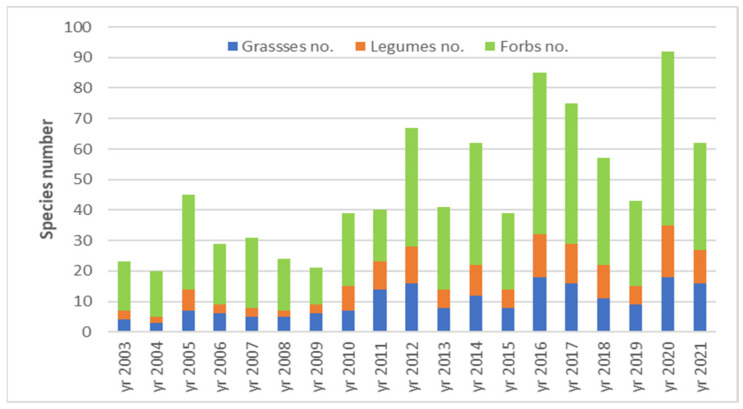
Dynamics of floristic composition of the main functional groups as species number (no.), during 2003–2021 period in ex-arable grassland from Grădinari.

**Figure 8:**
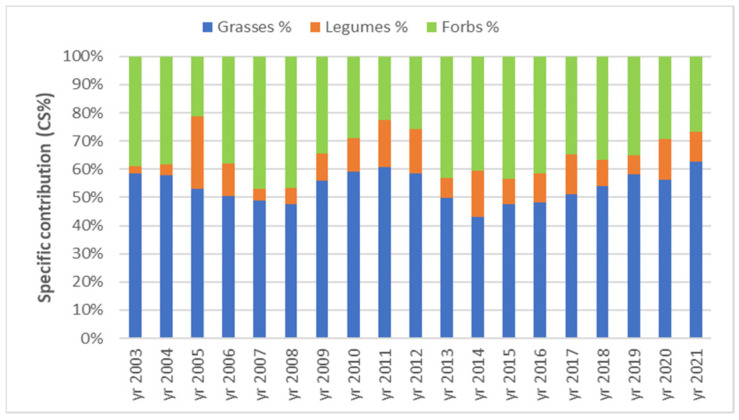
Dynamics of floristic composition of the main functional groups as specific contribution (CS%), during 2003–2021 period in ex-arable grassland from Grădinari.

The less numerous grass species were determined in 2004, namely three; and the greatest number of grass taxa was eighteen in the year 2020 (Figure 7). From the perspective of the specific contribution rate of the three functional groups, the differences were relatively small, because the lowest value was CS% = 43.06 in 2014 and the highest CS% = 62.77 in 2021 (Figure 8).

Air temperature correlated with floristic composition (Table 3) highlighted several significant results, namely some positive correlations in the case of grasses species number and the temperatures from March (*r* = 0.456 *) and from September (*r* = 0.505 *); and a negative one between the temperature from February and grasses CS% (*r* = −0.482 *). The other functional group correlated positively with the air temperature from February (*r* = 0.503 *) was forbs as species number. Between air temperature and legumes, functional group, correlations in the timeframe of the present research were not identified.

The species number of the main functional groups was not correlated with the rainfall amount during the experiment (Table 4), but the CS % values proved to be influenced by this climatic factor. Thus, the grasses’ contribution increases with the increase in the rainfalls from January (*r* = 0.549 *) and decreases with the increase in the rainfalls from March (*r* = −0.565 *). The rainfall amount proved to have a positive impact on the contribution rate of the legumes group (Table 4), namely for the rainfalls from April (*r* = 0.622 **) and from December (*r* = 0.592 **), in addition to the total rainfalls from the entire year (*r* = 0.531 *) and from the vegetation season (*r* = 0.637 **). Regarding the analysis of the potential influence of the rainfalls on the specific contribution of forbs, there was identified a positive correlation with the rainfalls from March (*r* = 0.553 *) and a negative correlation coefficient with the rainfalls from December (*r* = −0.465 *) (Table 4).

### 2.5. Pastoral Value Dynamics and Relationship with Air Temperature and Rainfall

**Figure 9:**
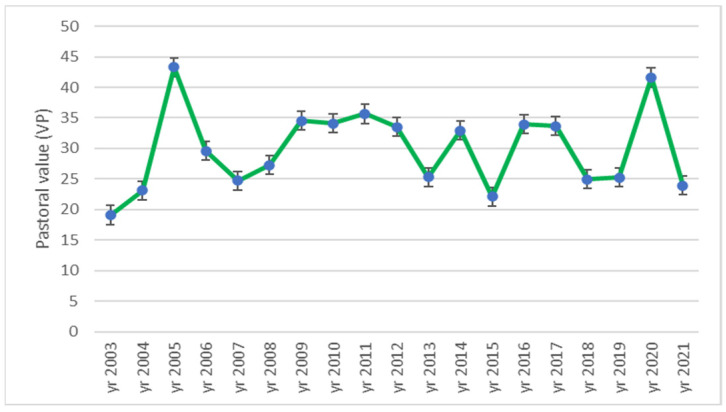
Dynamics of pastoral value (PV) during 2003–2021 period in ex-arable grassland from Grădinari (standard error displayed).

The pastoral value (PV) dynamic is displayed in Figure 9. The lowest pastoral value determined was PV = 19.09 in the year 2003, namely the first year of the investigation. The highest pastoral value was identified in 2005, namely PV = 43.31, closely followed by the PV from 2020, which was 41.6. At the beginning of the research, the PV was characteristic of the medium quality grassland (VP between 14 and 25). The highest values of the PV determined in the ex-arable successional grassland were characteristic for good pasture (PV greater than 25).

The only correlation identified between the air temperature analyzed values and the pastoral value was a negative one, namely for the temperature from June (*r* = −0.564 *), showing that the increase in the temperature from June determined the decrease in the pastoral value (Table 5).

The rainfalls have a greater impact on the pastoral value (Table 6) than do the temperatures (Table 5). There were identified a series of positive correlations: one highly significant in the case of the rainfall amount from the vegetation season (*r* = 0.716 ***), one very significant for the total annual rainfall amount (*r* = 0.692 **), and other significant correlations for some monthly rainfall amounts. Thus, the significant correlations of the pastoral value were with the rainfalls from the months of February (*r* = 0.475 *), June (*r* = 0.559 *), July (*r* = 0.469 *) and December (*r* = 0.475 *).

## 3. Discussion

### 3.1. Vegetation

Vegetation of a successional grassland has a dynamic structure [45,47,48,49,50]. Thus, the characterization of the sward with the phytosociological “*association*” concept is not always proper, because it characterizes the ecosystems found in the climax evolutional stage [51]. Secondary grasslands are managed agro-ecosystems, which can be characterized most properly [52] from a typological point of view [3,53] that covers more complex datasets relating to the vegetation with the stational conditions and with the management [54,55].

The random intervention with grazing cattle [35,49,56,57] and minimal maintenance works [3,58] influences intensively the grassland vegetation. The extensive grazing is a major source of propagules of grassland species, useful for the intensification of the succession process of the abandoned arable land to grassland.

The ex-arable grassland from Grădinari was extensively grazed by sheep since the abandonment, with the mention that in about the last 10 years it was grazed randomly. According with the literature, grazing is beneficial for ex-arable land succession to grassland [35]. Thus, the establishment of the grassland species during the succession seems to be mediated by the soil microbiota too [59], grazing being beneficial for the proliferation of the soil microorganisms specific for grassland [59,60,61], as they favor the establishment of the grazing tolerant species [61].

Random grazing with sheep [57,60,62] and mulching works [3,58] applied late in autumn could have an important contribution to the great restoration and high presence of late successional grassland species. The certain effect of the grazing can be considered the increase in the number of taxa (see Figure 2) in a significant manner.

Forrest steppe grassland is a notion referring to a vegetation type spread across Eurasia, fulfilling the criteria of a biome, with particularities that divide it in nine specific regions [45], our research area being framed in Region A: Southeast Europe from the geographical point of view. Some of the species identified in the experimental field characteristic for Region A were *Festuca valesiaca*, *Stipa capillata*, *Fragaria viridis*, *Brachypodium pinnatum*, etc. According to the classification on the regions proposed by Erdős et al. [45], some of the forest steppe grassland species identified in our experimental field are characteristic for other regions of this biome, namely for Region B: East Europe (*Filipnedula vulgaris*, *Salvia pratensis*), for Region D: West Siberia and North Kazahstan (*Lathyrus pratensis*, *Vicia cracca*) and for Region F: far East (*Botriochloa ischaemum*, *Poa pratensis*).

In the first year of vegetation records (2003) on the analyzed ex-arable grassland, numerous annual weed species were dominant, above all *Bromus hordeaceus*. However, at the same time, some perennial grasses such as *Lolium perenne*, *Calamagrostis epygeios* and *Cynodon dactylon* were identified (see Table A1). In 2006, *Festuca valesiaca* appeared in the sward, and since 2007 it became the most dominant species together with *Agrostis tenuis* as codominant, these species remaining the most abundant in all the years of the research. In other researches referring to the successional forest steppe grassland evolution from Hungary, *Calamagrostis epygeios* was an abundant species at the beginning of abandonment, but its cover decreased in the later abandonment phase [41,63] This species was not abundant in our analysed plot in general; even it was present there. This fact could be explained by a faster transition of the vegetation from the studied abandoned arable land to forest steppe grassland.

### 3.2. Climate

The evidence of the extreme climatic average values shows a great climate pressure, mainly in the case of the temperature increase that nowadays is a worrying phenomenon. The same trend was noticed in the studied area, with a multiannual normal value of the air temperature of 11.21 °C, where it registered the overpassing above the normal value in 18 years from all the 19 years of the research time interval. More than that, in some years, the yearly average value of the air temperature was even greater than 13 °C (in 2013, 2015, 2018 and 2019), the greatest registered average value being 13.70 °C in 2018, which represents a deviation of 2.49 °C, such extremes being mentioned in the literature referring to our region [36,64]. Thus, such extreme heat events were also registered in other neighbouring countries, such as Bulgaria [46].

The rainfall regime was characterized in general by extremes regarding the monthly distribution of the rainfalls during the year, with high amounts of rain in a very short time [65,66] or long time intervals of severe drought [67,68]. In the experimental area, the average rainfall amounts were in general near or above the multiannual averages, the situation being better in comparison with the temperatures that have strongly deviated from the average values.

In the analyzed time interval (2003–2021), the value of the main meteorological parameters implied in the development of the vegetation structure and typology had great variations. The general trend of the temperature, with few exceptions, was an increasing one since the beginning of the research time interval. In the case of rainfall amount, a general decrease in them was noticed toward to the end of the research interval, and an irregular variation from one year to the next. The Lang Rainfall Index multiannual mean value for the study area is 79.44, characteristic for warm temperate climate. The values of R had a great variation during 2003–2021 (R from 41.82 to 119.31). According to the characteristic classification of R [69], four years corresponded to the semiarid climate (years 2013, 2015, 2019 and 2021), fourteen years corresponded to the warm temperate climate and one year (2005) to the temperate humid climate. The Lang Rainfall Index will be analyzed in following research to see if the arid climate continues to occur in the studied area. The good transition of the analysed abandoned arable land to the secondary successional forest steppe grassland was probably influenced in a great measure by the relatively good rainfall regime at our experimental time scale.

### 3.3. Biodiversity Dynamics and Relationship with Air Temperature and Rainfall

The general evolution trend of the biodiversity analyzed as species richness, Shannon index and Simpson index highlighted a certain increase in the specific diversity of the analyzed ex-arable forest steppe grassland during the research interval. The evolution of species richness seemed to be influenced by year (see Figure 3). Such results regarding the increase in the species richness [4,5,30] in ex-arable forest steppe successional grassland and the Shannon and Simpson diversity indices [41,63] were found in the literature. Thus, often in practice the secondary succession of ex-arable grassland proved to determine the increase in the vegetation biodiversity [3,4,5,30,41,63,70], our results confirming this hypothesis for the analysed ex-arable forest steppe grassland in conditions of natural succession.

Climate influences grassland vegetation typology and structure [49,54] and the evolution by secondary succession of the abandoned arable land to grassland [42,45,47,71,72]. Air temperature proved to have an impact on biodiversity, there being determined the existence of some correlations, namely a positive one between the air temperature from February and the Shannon index (*r* = 0.479 *) and a negative one between the air temperature from September and the Simpson index (*r* = −0.498 *). There were noticed some other values close to the significant ones, which suggest the potential for the future investigation of this hypothesis. Extrapolating, the air temperature from February, March and September could also influence the increase in the biodiversity in the analyzed successional grassland, because the obtained *r_calc_* values were very close to the *r_critical_* values. The rainfall amounts from the experimental area seemed to not be very influential for biodiversity, from statistical point of view, during the succession at the time scale of our experiment. Some of the researches regarding the impact of climate change on grassland vegetation biodiversity assumes the existence of at least a partial influence of the climatic extremes [67,73,74,75]. Aside from the analyzed climatic factors, other factors were implied probably too, with stronger influence on the biodiversity, such as random grazing with sheep and mulching works applied late in autumn. They could have an important contribution to the great natural restoration process and even an increase in the biodiversity, such results being confirmed by the literature [28,49,58,60,70].

### 3.4. Florisic Composition Dynamics and Relationship with Air Temperature and Rainfall

Floristic composition is a feature of the grassland vegetation community influenced by the cumulated actions of the biotic and abiotic factors with the management [39,45,47,49]. The dominance of the perennial grasses in the sward of the analyzed ex-arable successional grassland is a good sign of the vegetation recovery [3,4,76]. Even the statistical results regarding the influence of the air temperature on grassland species were less statistically significant at our experiment time scale. The appearance in 2015 of the thermophilic and xerophytic grass species *Stipa capillata* and its constant presence in the following years of the research suggest the adaptation of the vegetation sward to dryer and hotter summers. The negative correlation of the grasses’ contribution rate with the rainfalls from March could be explained by the low soil temperatures and nebulosity, which could affect the grasses’ growth at the beginning of spring.

The establishment of the perennial grasses in the abandoned arable land contributes to the succession of the secondary grassland vegetation, and the climate can be a driver in this way together with the management [28,60,73,76].

Legumes are also an important element of the quality of the grassland vegetation [13,43,60]. In the present experiment, it was noticed that they were the most sensitive to the rainfall regime. An interesting situation was noticed in the case of *Trifolium alexandrinum*, a Mediterranean species that occurred in a relatively high rate (CS% = 9.71) in the year 2005. This species’ propagules were probably brought by the sheep that have probably grazed previously on a plot abundant in *T. alexandrinum* from the farm, where it was seeded in the 1960s and is still abundant and self-seeding since then. The presence of *T. alexandrinum* is not constant in the experimental plot; the next appearances were registered in rates comprised between 0.38 and 1.44% in the years 2010–2012, 2016–2017 and 2020. The high rate of *T. alexandrinum* from 2005 could be explained by the numerous empty ecological niches of the ex-arable grassland in the early successional phase that are decreasing while the succession process is advancing.

Thus, the air temperature seemed to not be correlated with the contribution rate of the legumes at the timescale of the present research. However, in the case of the rainfall correlation with the specific contribution of the legumes, the results obtained highlighted that this functional group was positively influenced by the yearly rainfall regime (RA I–XII) and by the rainfall regime from the vegetation period (RA III–IX). Results highlighting the impact of climate on the functional groups were investigated by the modeling and creating of algorithms, which can have future application on the future research of the temperate grasslands [73].

The presence of the forbs is an important element of the grassland floristic composition [43,76,77]. They are bringing added value by certain valuable species from other botanical families rich in nutritive substances and active compounds (e.g., *Achillea millefolium*, *Cichorium intybus*, *Plantago* sp., *Taraxacum officinale*, etc.) and are enriching the biodiversity [54,78]. A balanced floristic composition is essential for the stability of the grassland ecosystem and for its resilience in the situation of unfavorable conditions such as extreme weather or inappropriate management (e.g., overgrazing, under-grazing, abandonment etc.) [28,45,63]. In a recent research, the hypothesis regarding the interaction between the grassland functional groups of plants and climate, together with other biotic and abiotic data, was used in the development of a modeling code in Norway for alpine grassland with the purpose to be used in the future as a tool in grassland ecosystem analysis [77].

### 3.5. Pastoral Value Dynamics and Relationship with Air Temperature and Rainfall

The evolution of the PV during the research was very irregular, but the greatest increase was noticed in the third year of research, namely the eleventh year after the land abandonment. According to the literature, in Alta Murgia National Park from Southern Italy, the pastoral value of the successional grasslands from arable land abandoned by more than 50 years was higher even in comparison with the semi-natural grasslands [79].

The impact of the air temperature on the pastoral value was not concluded during the experiment; the only correlation identified showed the decrease in the PV due to the increase in the air temperature from June (*r* = −0.564 *). The rainfall regime during the entire year and during the vegetation season seemed to have the most significant impact on the pastoral value of the studied successional forest steppe grassland from the experiment (*r* = 0.692 **; *r* = 0.716 ***). In the literature, there are not numerous the references regarding the influence of the temperature and rainfall amount on the pastoral value of successional grasslands for such timeframes, but some were focused on the influence of rainfall on dry matter yield [47] and fresh fodder yield [73]. Thus, the results obtained by Tripolskaya et al. [28] highlighted the small impact of the climate change on the temperate grassland yield due to the early spring water supply that mitigates the later rainfall deficit. Our monthly results suggested the same thing, namely the significant positive correlations between the rainfall amount from December (*r* = 0.475 *) and from February (*r* = 0.475 *) on the pastoral value (see Table 6), but in our case the rainfalls from June (*r* = 0.559 *) and July (*r* = 0.469 *) had a positive influence on the PV too.

## 4. Materials and Methods

### 4.1. Description of the Study Area

**Figure 10:**
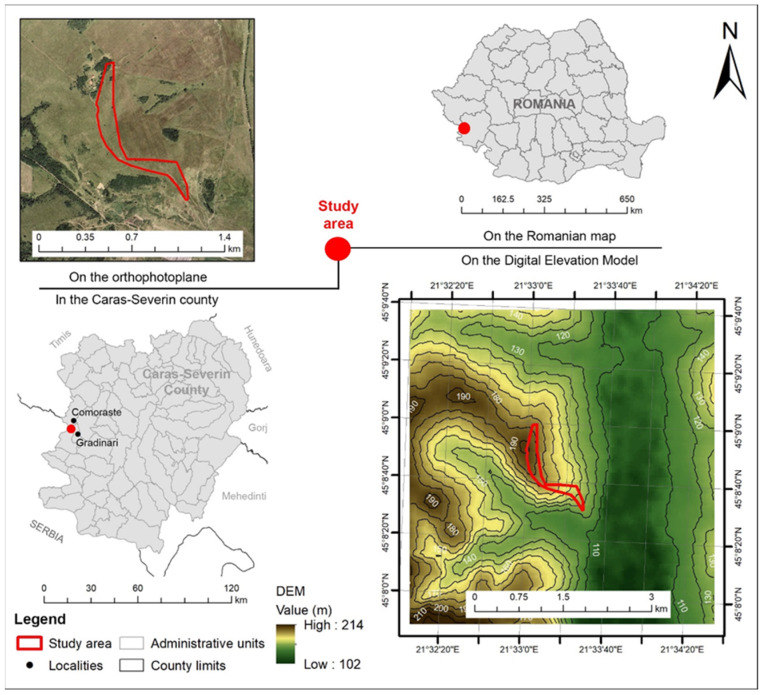
Localization of the study area (designed by Copăcean L. using data from Geo-spatial.org) [80], ANCPI [81], EEA–EU-DEM [82].

The background of the present research concerns the investigation of the climate implications for the natural recovery of an ex-arable forest steppe grassland. The vegetation data were collected from the Mezdraia area belonging to the locality of Grădinari (Caraş-Severin County, Western Romania) from an ex-arable grassland plot (Figure 10).

**Figure 11:**
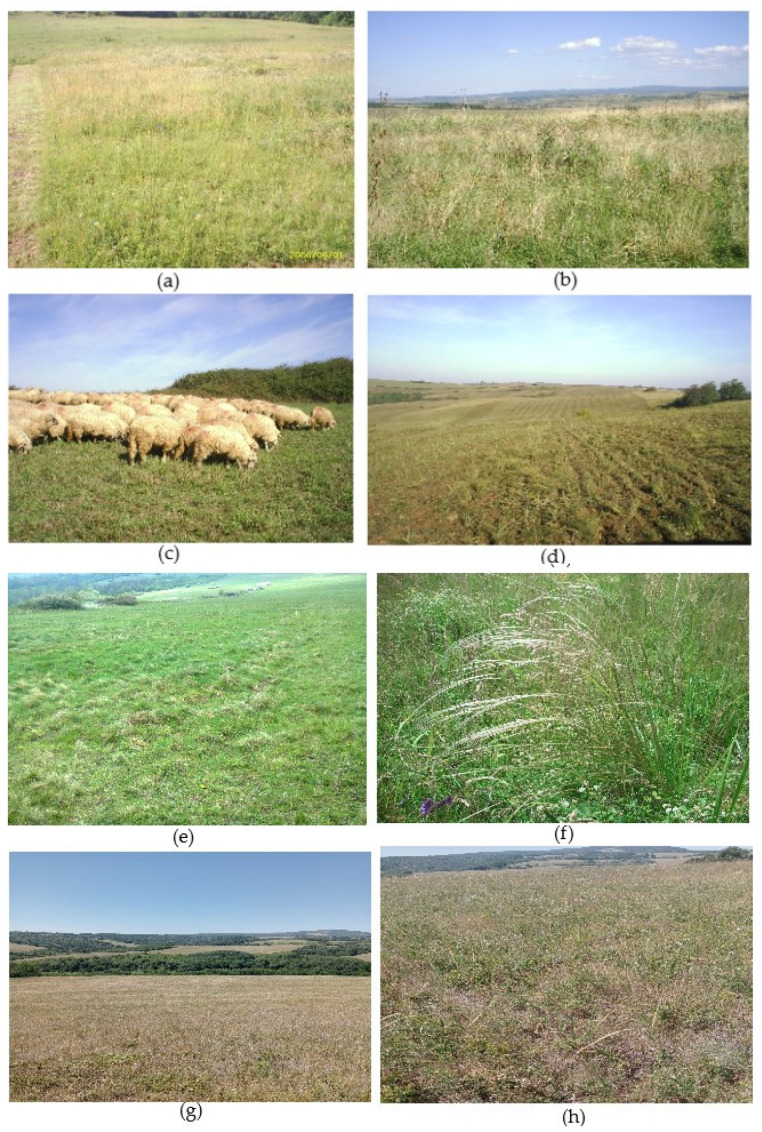
Successional ex-arable grassland from Grădinari: (**a**) general view from 2006; (**b**) general view from 2007; (**c**) sheep grazing in 2007; (**d**) after mulching in 2007; (**e**) view from 2012; (**f**) *Stipa capillata* in 2015; (**g**,**h**) view from 2021 [photo: Sărățeanu V.].

The study area is located in the southwest part of Romania, on the territory of Caraş-Severin County, at a distance of 2 km from the border with the Republic of Serbia. The experimental site (45°8′40″ N, 21°33′20″ E) has a surface of 10 hectares and is located in the Tirolului Hills. According to the data obtained by the processing of the Digital Elevation Model (DEM) [82] using methods and techniques specific for the Geographic Informatic Systems (GIS) [83], it was noticed that the experimental site is placed at elevations comprising 127–191 m, with the average altitude of 170 m a.s.l. Because the area is hilly (see Figure 10), the slope varies between 2.4 and 16.3%, with an average value of 9.4%. The experimental site is placed on the eastern and southeastern slope, which influences the vegetation type. From the biogeographical point of view, the experimental site belongs to the forest steppe area [84,85]. Figure 11 presents some views from the analyzed field at different moments during the research.

The biogeographical vegetation framing of the studied area is forest steppe and the soil type is brown forest soil [86]. The arable land field has been abandoned since the year 1995 [86] and the vegetation has evolved gradually by natural succession. Since the abandonment, the analyzed successional grassland surface has been used by sheep for random extensive grazing. The maintenance work applied was only mulching, which occurred late in autumn at the ending of grazing season, but it is also applied randomly.

### 4.2. Vegetation Data Collection and Analysis

The vegetation data were collected for 19 years, namely from the year 2003 to 2021 (months May, July and September). The vegetation surveys were conducted with the linear point quadrate method described by Daget-Poissonet [87]. The considered vegetation features in the present research were the following: floristic composition (grasses, legumes and forbs considered as species number and specific contribution—CS%) [3,39,47,48,77,88], biodiversity (as species richness—S, Shannon index—H′ [3,89,90] and Simpson index (D) [3,48,91]) and pastoral value—VP (0–100 scale) [3,53,87,92]. The pastoral value characterizes the relative forager value of the grassland sward based on the specific contribution in the grassland cover of the species with the forager value expressed as the Specific Forage Quality Index (SFQi) [78,87]. The Specific Forage Quality Index (SFQi) used for the VP calculation is characteristic for the Romanian grassland species [78].

### 4.3. Climate Data Collection and Analysis

The multiannual average temperature in the study area is 11.21 °C, and multiannual average rainfall amount is 890.5 mm. The climate parameters considered in this work were air temperature and rainfall amount registered at the nearest meteorological station (Oraviţa, Caraş-Severin County). The climate data used in statistical analyses were grouped in climatic years (months I–XII), vegetation seasons (months III–IX) and monthly averages.

The Lang Rainfall Index (R) represents a ratio between the rainfall amount and air temperature [69,93,94], namely the “connection” between the two analyzed climate parameters, because temperature is considered one of the main factors implied in the process of evapotranspiration [95]. The Lang Rain Factor is also named the pluvial-thermal index [95] and illustrates the water inputs and outputs from a territory. It was taken into consideration because it is considered to be influential for the structure and typology of vegetation.

### 4.4. Statistical Analysis

Based on the vegetations and climate data, a series of statistical analyses were undertaken. The software programs used for data processing were Excel 2019 [96] and JASP 0.16.4.0; the statistical methods used were Pearson *r* [97,98,99] and a multinomial test [99].

The aim of this research was to release new outputs and datasets regarding the evolution of the vegetation sward of successional ex-arable grassland from the forest steppe area related to air temperature and rainfall amounts in conditions of natural succession during almost two decades, namely nineteen years (time interval 2003–2021).

## 5. Conclusions

Successional grasslands are a potential resource of biomass, biodiversity and habitat for wildlife. Such naturally recovered ex-arable grassland ecosystems are able to restore their natural cycles and provide ecological and socio-economical services as a natural ecosystem.

The resilience of the analyzed ex-arable land was good even in the present climate conditions, with years characterized by higher temperatures and severe droughts or hard rainy time intervals. The rainfall regime less disturbed in comparison with the temperature probably had a good impact on the transition of the abandoned arable land to successional forest steppe grassland at our experiment time scale. However, the appearance of the xerophytic species *Stipa capillata* since 2015 could be connected with the appearance of the years with a semiarid climate (years 2013, 2015, 2019 and 2021). This trend should be investigated in future research to check if the hypothesis of climate warming is confirmed.

The other element that has probably contributed to the good recovery of the grassland vegetation could be soil seed bank. However, the random grazing with sheep and mulching works applied late in autumn could have a very important contribution to the great natural restoration process and even on the increase in the biodiversity and the high presence of late successional grassland species. The domestic and wild herbivores, namely the grassland maintenance tools and machineries, probably were vectors for some grassland species’ propagules that probably have speed up the natural restoration process of the grassland species. Thus, climate change pressure on the natural restoration of the biodiversity and pastoral value of ex-arable forest steppe grassland could be mitigated at least partially by random grazing and mulching works.

The outputs from this work can be helpful for the setting of strategies of natural recovery of great surfaces of abandoned fields from the forest steppe area in the context of climate change. The impact of climate change on the natural restoration of ex-arable successional grassland should be investigated more for a better understanding of the implications at different levels such as vegetation structure, biodiversity, floristic composition, forage value, productivity, carbon sequestration, economic and social value, etc.

## Figures and Tables

**Table 1:** Correlations between the averages of the temperature (I–XII) and species biodiversity (S, H′ and D) in ex-arable grassland from Grădinari (period 2003–2021).

Variable		I	II	III	IV	V	VI	VII	VIII	IX	X	XI	XII	I–XII	III–IX
S	Pearson’s *r*	−0.029	0.455	0.436	0.075	−0.333	0.072	0.053	−0.052	0.351	−0.026	−0.158	−0.050	0.204	0.175
	*p*-value	0.908	0.050	0.062	0.759	0.163	0.771	0.828	0.831	0.141	0.917	0.518	0.838	0.401	0.473
H′	Pearson’s *r*	0.021	**0.479** *	0.449	0.251	−0.290	0.102	0.107	0.056	0.442	0.001	−0.090	−0.089	0.338	0.323
	*p*-value	0.932	0.038	0.054	0.300	0.229	0.678	0.662	0.819	0.058	0.997	0.714	0.718	0.157	0.178
D	Pearson’s *r*	−0.106	−0.393	−0.368	−0.366	0.241	−0.146	−0.073	−0.131	**−0.498** *	−0.023	0.063	0.061	−0.398	−0.396
	*p*-value	0.664	0.096	0.121	0.123	0.320	0.550	0.767	0.592	0.030	0.924	0.798	0.805	0.091	0.093

* *p* < 0.05.

**Table 2:** Correlations between the rainfall amounts (I–XII) and species biodiversity (S, H′ and D) in ex-arable grassland from Grădinari (period 2003–2021).

Variable		I	II	III	IV	V	VI	VII	VIII	IX	X	XI	XII	I–XII	III–IX
S	Pearson’s *r*	−0.089	−0.096	−0.166	−0.030	0.302	0.293	0.293	0.017	0.077	−0.011	−0.372	0.031	0.171	0.311
	*p*-value	0.716	0.695	0.498	0.902	0.209	0.223	0.224	0.945	0.754	0.963	0.117	0.899	0.483	0.195
H′	Pearson’s *r*	−0.210	−0.042	−0.067	−0.021	0.345	0.339	0.259	−0.028	0.024	−0.116	−0.397	0.118	0.166	0.327
	*p*-value	0.389	0.863	0.786	0.931	0.148	0.156	0.285	0.910	0.922	0.635	0.093	0.630	0.498	0.172
D	Pearson’s *r*	0.155	−0.029	0.024	−0.021	−0.410	−0.296	−0.264	0.098	0.015	0.191	0.410	−0.279	−0.193	−0.322
	*p*-value	0.526	0.905	0.921	0.930	0.082	0.218	0.274	0.691	0.951	0.434	0.081	0.247	0.429	0.179

**Table 3:** Correlations between the averages of the temperature (I–XII) and floristic composition in ex-arable grassland from Grădinari (period 2003–2021).

Variable		I	II	III	IV	V	VI	VII	VIII	IX	X	XI	XII	I–XII	III–IX
Grasses no.	Pearson’s *r*	−0.068	0.363	**0.456** *	−0.019	−0.366	0.108	0.087	0.029	**0.505** *	−0.111	−0.208	0.058	0.188	0.219
	*p*-value	0.783	0.127	0.050	0.938	0.123	0.659	0.724	0.907	0.028	0.651	0.393	0.814	0.441	0.368
Legumes no.	Pearson’s *r*	−0.048	0.346	0.361	0.102	−0.298	0.114	−0.017	2.799 × 10^−4^	0.438	−0.078	−0.147	−0.007	0.189	0.210
	*p*-value	0.844	0.147	0.129	0.678	0.216	0.642	0.944	0.999	0.061	0.751	0.547	0.978	0.439	0.387
Forbs no.	Pearson’s *r*	−0.005	**0.503** *	0.430	0.098	−0.314	0.039	0.062	−0.099	0.242	0.027	−0.134	−0.104	0.204	0.136
	*p*-value	0.984	0.028	0.066	0.689	0.191	0.875	0.801	0.687	0.319	0.914	0.585	0.672	0.402	0.577
Grasses %	Pearson’s *r*	−0.392	**−0.482** *	−0.151	−0.210	−0.162	0.123	−0.179	0.133	0.262	−0.122	0.108	0.371	−0.205	−0.041
	*p*-value	0.097	0.037	0.538	0.388	0.508	0.616	0.462	0.586	0.278	0.618	0.660	0.117	0.401	0.869
Legumes %	Pearson’s *r*	−0.118	−0.165	−0.133	−0.056	−0.350	−0.295	−0.080	−0.428	0.319	−0.111	−0.214	0.037	−0.341	−0.285
	*p*-value	0.629	0.500	0.586	0.821	0.142	0.220	0.744	0.068	0.184	0.650	0.379	0.880	0.153	0.238
Forbs %	Pearson’s *r*	0.359	0.455	0.201	0.187	0.363	0.125	0.183	0.212	−0.411	0.165	0.077	−0.287	0.387	0.232
	*p*-value	0.131	0.050	0.410	0.443	0.127	0.611	0.454	0.384	0.081	0.500	0.754	0.234	0.102	0.340

* *p* < 0.05.

**Table 4:** Correlations between the rainfall amounts (I–XII) and floristic composition in ex-arable grassland from Grădinari (period 2003–2021).

Variable		I	II	III	IV	V	VI	VII	VIII	IX	X	XI	XII	I–XII	III–IX
Grasses no.	Pearson’s *r*	−0.010	−0.175	−0.198	−0.094	0.256	0.193	0.350	−0.140	−0.128	−0.029	−0.325	0.044	0.035	0.149
	*p*-value	0.968	0.473	0.416	0.702	0.290	0.429	0.141	0.567	0.602	0.906	0.175	0.858	0.886	0.542
Legumes no.	Pearson’s *r*	−0.052	−0.064	−0.216	−0.065	0.342	0.307	0.418	−0.086	−0.024	−0.041	−0.408	0.143	0.180	0.300
	*p*-value	0.831	0.795	0.374	0.791	0.152	0.202	0.075	0.725	0.921	0.869	0.083	0.561	0.460	0.211
Forbs no.	Pearson’s *r*	−0.127	−0.071	−0.126	0.008	0.289	0.310	0.211	0.112	0.187	0.006	−0.356	−0.014	0.211	0.359
	*p*-value	0.603	0.771	0.606	0.975	0.230	0.196	0.387	0.647	0.444	0.980	0.135	0.955	0.386	0.131
Grasses %	Pearson’s *r*	**0.549** *	−0.086	**−0.565** *	−0.152	0.173	−0.145	0.253	−0.368	−0.453	0.136	−0.034	0.060	−0.192	−0.335
	*p*-value	0.015	0.727	0.012	0.535	0.479	0.554	0.297	0.121	0.051	0.579	0.890	0.806	0.430	0.160
Legumes %	Pearson’s *r*	−0.299	0.294	−0.220	**0.622** **	0.295	0.188	0.375	0.379	0.108	−0.250	−0.448	**0.592** **	0.531 *	**0.637** **
	*p*-value	0.214	0.222	0.364	0.004	0.219	0.441	0.114	0.110	0.661	0.302	0.055	0.008	0.019	0.003
Forbs %	Pearson’s *r*	−0.171	−0.150	**0.553** *	−0.337	−0.332	−0.032	−0.444	−0.013	0.240	0.083	0.343	**−0.465** *	−0.244	−0.220
	*p*-value	0.484	0.541	0.014	0.158	0.165	0.895	0.057	0.958	0.322	0.734	0.150	0.045	0.314	0.366

* *p* < 0.05, ** *p* < 0.01.

**Table 5:** Correlations between the averages of the temperature (I–XII) and pastoral value (PV) in ex-arable grassland from Grădinari (period 2003–2021).

Variable		I	II	III	IV	V	VI	VII	VIII	IX	X	XI	XII	I–XII	III–IX
PV	Pearson’s *r*	−0.253	9.629 × 10^−4^	−0.072	0.181	−0.412	**−0.564** *	−0.159	−0.436	0.280	−0.139	−0.241	−0.008	−0.374	−0.331
	*p*-value	0.296	0.997	0.770	0.459	0.079	0.012	0.515	0.062	0.245	0.569	0.320	0.973	0.114	0.166

* *p* < 0.05

**Table 6:** Correlations between the rainfall amounts (I–XII) and pastoral value (PV) in ex-arable grassland from Grădinari (period 2003–2021).

Variable		I	II	III	IV	V	VI	VII	VIII	IX	X	XI	XII	I–XII	III–IX
PV	Pearson’s *r*	−0.425	**0.473** *	−0.247	0.390	0.136	**0.559** *	**0.469** *	0.352	0.163	0.095	−0.322	**0.475** *	**0.692** **	**0.716** ***
	*p*-value	0.069	0.041	0.307	0.099	0.578	0.013	0.043	0.139	0.505	0.699	0.178	0.040	0.001	<0.001

* *p* < 0.05, ** *p* < 0.01, *** *p* < 0.001.

## Data Availability

Not applicable.

## Appendix A

**Table A1:** Floristic composition of the ex-arable successional forest steppe grassland from Grădinari (Caraș-Severin County) based on the main grassland species functional groups (grasses, legumes and forbs) during 2003–2021.

Year	Grasses	Legumes	Forbs
2003	*Bromus hordeaceus; Calamagrostis epigeios; Cynodon dactylon; Lolium perenne*	*Dorycnium pentaphyllum; Lotus corniculatus; Trifolium arvense*	*Agrimonia eupatoria; Anthemis arvensis; Aster linosyris; Centaurea jacea; Cirsium arvense; Convolvulus arvensis Erygeron anuus; Filago arvensis; Geranium pusillum; Leontodon autumnalis; Plantago media; Potentilla erecta; Rubus caesius; Senecio vulgaris; Sonchus asper; Viola tricolor*
2004	*Apera spica-venti; Bromus hordeaceus; Lolium perenne*	*Dorycnium pentaphyllum; Lotus corniculatus*	*Agrimonia eupatoria; Anthemis arvensis; Centaurea jacea; Cirsium arvense; Convolvulus arvensis; Filago arvensis; Erygeron anuus; Geranium pusillum; Leontodon autumnalis; Plantago media; Potentilla erecta; Rubus caesius; Senecio vulgaris; Sonchus asper; Viola tricolor*
2005	*Agrostis tenuis; Apera spica-venti; Bromus hordeaceus; Festuca valesiaca; Holcus lanatus; Lolium perenne; Poa pratensis*	*Dorycnium pentaphyllum; Lathyrus tuberosus; Lotus corniculatusl Medicago lupulina; Trifolium arvense; Trifolium alexandrinum; Vicia cracca*	*Achillea millefolium; Agrimonia eupatoria; Aristolochia clematitis; Artemisia austriaca; Carthamus lanatus; Cichorium intybus; Cirsium arvense; Convolvulus arvensis; Dianthus carthusianorum; Echium vulgare; Erygeron anuus; Eryngium campestre; Euphorbia cyparissias; Filipendula vulgaris; Fragaria viridis; Galium verum; Geranium pusillum; Hypericum perforatum; Mentha arvensis; Origanum vulgare; Plantago lanceolata; Plantago media; Potentilla erecta; Prunella vulgaris; Rubus caesius; Rumex crispus; Scabiosa ochroleuca; Tanacetum vulgaris; Taraxacum officinalis; Thymus serpyllum. Verbena officinalis*
2006	*Agrostis tenuis; Bromus hordeaceus; Festuca valesiaca; Holcus lanatus; Lolium perenne; Poa pratensis*	*Lotus corniculatus; Medicago lupulina; Trifolium arvense; Trifolium repens*	*Achillea millefolium; Agrimonia eupatoria; Anthemis arvensis, Cichorium intybus; Convolvulus arvensis; Erygeron anuus; Filipendula vulgaris; Geranium pusillum; Gypsophila muralis; Leontodon autumnalis; Plantago lanceolata; Plantago major; Plantago media; Potentilla erecta; Prunella vulgaris; Rubus caesius; Tanacetum vulgaris; Taraxacum officinalis; Thymus serpyllum;*
2007	*Agrostis tenuis; Bromus hordeaceus; Festuca valesiaca; Holcus lanatus; Poa pratensis*	*Dorycnium pentaphyllum; Lotus corniculatus; Medicago lupulina;*	*Achillea millefolium; Aster linosyris; Carthamus lanatus; Centaurea jacea; Cichorium intybus; Cirsium arvense; Convolvulus arvensis; Crepis biennis; Daucus carota; Erygeron anuus; Euphorbia cyparissias; Filipendula vulgaris; Fragaria viridis; Galium verum; Geranium pusillum; Hieracium pilosella; Linaria vulgaris; Plantago lanceolata; Plantago media; Prunella vulgaris; Rumex crispus; Senecio jacobea; Thymus serpyllum*
2008	*Agrostis tenuis; Bromus hordeaceus; Festuca valesiaca; Holcus lanatus; Poa pratensis*	*Dorycnium pentaphyllum; Lotus corniculatus; Prunella vulgaris; Thymus serpyllum*	*Achillea millefolium; Aster linosyris; Cichorium intybus; Convolvulus arvensis; Crepis biennis; Daucus carota; Erygeron anuus; Euphorbia cyparissias; Filipendula vulgaris; Fragaria viridis; Galium verum; Geranium pusillum; Hieracium pilosella; Plantago lanceolata; Plantago media; Prunella vulgaris; Thymus serpyllum*
2009	*Agrostis tenuis; Briza media; Festuca valesiaca; Holcus lanatus; Lolium perenne; Poa pratensis;*	*Lotus corniculatus; Trifolium arvense; Vicia cracca*	*Achillea millefolium; Agrimonia eupatoria; Convolvulus arvensis; Conyza canadensis; Eryngium campestre; Euphorbia cyparissias; Filipendula vulgaris; Geranium pusillum; Gypsophylla muralis; Potentilla erecta; Rubus caesius; Stachys officinalis*
2010	*Agrostis tenuis; Bromus hordeaceus; Calamagrostis epigeios; Festuca valesiaca; Holcus lanatus; Poa pratensis; Setaria viridis*	*Dorycnium pentaphyllum; Lathyrus pratensis; Lotus corniculatus; Trifolium arvense; Trifolium dubium; Trifolium alexandrinum; Trifolium pannonicum; Trifolium repens*	*Achillea millefolium; Agrimonia eupatoria; Aster linosyris; Centaurea jacea; Convolvulus arvensis; Crepis biennis; Cychorium intybus; Daucus carota; Dianthus cartusianorum; Erygeron anuus; Filipendula vulgaris; Fragaria viridis; Geranium pusillum; Hieracium pilosella; Inula britannica; Leontodon autumnalis; Plantago lanceolata; Plantago media; Potentilla erecta; Rubus caesius; Senecio jacobaea; Stachys officinallis; Taraxacum officinale; Verbena officinallis*
2011	*Agropyron repens; Agrostis tenuis; Alopecurus pratensis; Anthoxanthum odoratum; Brachipodium pinnatum; Briza media; Bromus hordeaceus; Calamagrostis epigeios; Cynosurus cristatus; Festuca rupicola; Festuca valesiaca; Holcus lanatus; Lolium perenne; Poa pratensis*	*Dorycnium pentaphyllum; Lathyrus nissolia; Lotus corniculatus; Medicago lupulina; Trifolium alexandrinum; Trifolium pratense; Trifolium repens; Vicia cracca; Vicia grandiflora*	*Achillea millefolium; Carex vulpina, Centaurea jacea, Cirsium arvense; Clematis integrifolia; Crepis biennis, Eryngium campestre; Filipendula vulgaris; Fragaria viridis; Galium mollugo; Galium verum; Knautia arvensis; Mentha arvensis; Plantago lanceolata; Potentilla erecta; Rosa gallica; Scabiosa ochroleuca*
2012	*Agropyron repens; Agrostis tenuis; Alopecurus pratensis; Anthoxanthum odoratum; Botriochloa ischaemum; Brachipodium pinnatum; Briza media; Bromus hordeaceus; Calamagrostis epigeios; Cynosurus cristatus; Festuca rupicola; Festuca valesiaca; Holcus lanatus; Lolium perenne; Poa pratensis; Sieglingia decumbens;*	*Dorycnium pentaphyllum; Lathyrus nissolia; Lathyrus pratense; Lathyrus tuberosus; Lotus corniculatus; Medicago lupulina; Trifolium alexandrinum; Trifolium pratense; Trifolium repens; Vicia cracca; Vicia grandiflora; Vicia tetrasperma*	*Achillea millefolium; Allium flavum; Aster linosyris; Carex vulpine; Centaurea jacea; Centaurium erythraea; Chrisanthemum leucanthemum; Cirsium arvense; Clematis integrifolia; Crepis biennis; Erygeron anuus; Eryngium campestre; Euphorbia cyparissias; Euphorbia seguieriana; Filipendula vulgaris; Fragaria viridis; Galium mollugo; Galium verum, Geranium pusillum; Inula britannica; Knautia arvensis; Linaria vulgaris; Marrubium vulgare; Mentha arvensis, Ornithogallum umbellatum; Plantago lanceolata; Plantago media; Potentilla erecta; Ranunculus acris; Rosa gallica, Scabiosa ochroleuca; Stellaria graminea; Taraxacum officinalis; Thalictrum aquilegifolium; Thymus serpyllum; Tragopogon arvensis; Veronica chamaedrys; Veronica spicata; Xeranthemum inapertum;*
2013	*Agrostis tenuis; Agropyron repens; Botriochloa ischaemum; Bromus hordeaceus; Calamagrostis epigeios; Festuca valesiaca; Holcus lanatus; Poa pratensis;*	*Dorycnium pentaphyllum; Lotus corniculatus; Medicago lupulina; Trifolium pratense; Trifolium repens; Vicia grandiflora*	*Achillea millefolium; Aster linosyris; Carthamus lanatus; Centaurea jacea; Cichorium intybus; Cirsium arvense; Convolvulus arvensis; Crepis biennis; Daucus carota; Erygeron anuus; Euphorbia cyparissias; Filipendula vulgaris; Fragaria viridis; Galium verum; Geranium pusillum; Hieracium pilosella; Linaria vulgaris; Marrubium vulgare; Ornithogallum umbellatum; Plantago lanceolata; Plantago media; Prunella vulgaris; Rumex crispus; Senecio jacobea; Thymus serpyllum; Rosa gallica; Xeranthemum inapertum*
2014	*Agropyron repens; Agrostis tenuis; Brachipodium pinnatum; Briza media; Bromus hordeaceus; Calamagrostis epigeios; Cynosurus cristatus; Dactylis glomerata; Festuca valesiaca; Holcus lanatus; Poa pratensis; Sieglingia decumbens*	*Allium flavum; Aster linosyris; Dorycnium pentaphyllum; Lathyrus pratensis; Lotus corniculatus; Medicago lupulina; Trifolium arvense; Trifolium pannonicum; Trifolium repens; Vicia cracca; Vicia grandiflora; Vicia tetrasperma*	*Achillea millefolium; Agrimonia eupatoria; Carex vulpina; Centaurium erythraea; Centaurea jacea; Crepis biennis; Cychorium intybus; Daucus carota; Dianthus carthusianorum; Euphrasia stricta; Filipendula vulgaris; Fragaria viridis; Galium mollugo; Galium verum; Geranium pusillum; Gypsophylla muralis; Hieracium pilosella; Inula britannica; Leontodon autumnalis; Plantago lanceolata; Plantago media; Potentilla erecta; Prunella vulgaris; Rosa gallica; Rubus caesius; Scabiosa ochroleuca; Senecio jacobaea; Stachys officinallis; Stellaria graminea; Taraxacum officinalis; Thalictrum aquilegifolium; Thymus serpyllum; Tragopogon orientalis; Verbena officinallis; Veronica chamaedrys; Veronica spicata; Verbena officinallis; Xeranthemum inapertum*
2015	*Agrostis tenuis; Agropyron repens; Botriochloa ischaemum; Bromus hordeaceus; Calamagrostis epigeios; Festuca valesiaca; Poa pratensis; Stipa capillata*	*Dorycnium pentaphyllum; Lotus corniculatus; Medicago lupulina; Trifolium pratense; Trifolium repens; Vicia grandiflora*	*Achillea millefolium; Aster linosyris; Carthamus lanatus; Centaurea jacea; Cichorium intybus; Crepis biennis; Daucus carota; Erygeron anuus; Euphorbia cyparissias; Filipendula vulgaris; Fragaria viridis; Galium verum; Geranium pusillum; Hieracium pilosella; Linaria vulgaris; Marrubium vulgare; Plantago lanceolata; Plantago media; Prunella laciniata; Prunella vulgaris; Rumex crispus; Senecio jacobea; Thymus serpyllum; Rosa gallica; Xeranthemum inapertum*
2016	*Agropyron repens; Agrostis stolonifera; Agrostis tenuis; Alopecurus pratensis; Anthoxanthum odoratum; Botriochloa ischaemum; Brachipodium pinnatum; Briza media; Bromus hordeaceus; Calamagrostis epigeios; Cynosurus cristatus; Dactylis glomerata; Festuca rupicola; Festuca valesiaca; Holcus lanatus; Lolium perenne; Poa pratensis; Sieglingia decumbens*	*Dorycnium pentaphyllum; Genista tinctoria; Lathyrus nissolia; Lathyrus pratense; Lathyrus tuberosus; Lotus corniculatus; Medicago lupulina; Ononis spinosa; Trifolium alexandrinum; Trifolium pannonicum; Trifolium pratense; Trifolium repens; Vicia cracca; Vicia grandiflora*	*Achillea millefolium; Agrimonia eupatoria; Allium flavum; Allium vineale; Aster linosyris; Campanula rapunculus; Carex vulpina; Centaurea jacea; Centaurium erythraea; Cerastium arvense; Cichorium intybus; Crepis biennis; Cirsium arvense; Clematis integrifolia; Crepis biennis; Daucus carota; Dianthus carthusianorum; Erygeron anuus; Eryngium campestre; Euphrasia stricta; Euphorbia cyparissias; Falcaria vulgaris; Filipendula vulgaris; Fragaria viridis; Galium mollugo; Galium verum; Geranium pusillum; Hieracium pilosella; Hypericum perforatum; Knautia arvensis; Leonthodon autumnalis; Linaria vulgaris; Luzula arvensis; Mentha arvensis; Origanum vulgare; Picris echioides; Plantago lanceolata; Polygala vulgaris; Potentilla erecta; Prunella laciniata; Prunella vulgaris; Rosa gallica; Rumex acetosella; Rumex crispus; Scabiosa ochroleuca; Senecio jacobea; Tanacetum vulgare; Stellaria graminea; Tragopogon orientalis; Verbena officinallis; Veronica chamaedrys; Veronica hederifolia; Veronica spicata*
2017	*Agropyron repens; Agrostis tenuis; Alopecurus pratensis; Anthoxanthum odoratum; Botriochloa ischaemum; Brachipodium pinnatum; Briza media; Bromus hordeaceus; Calamagrostis epigeios; Cynosurus cristatus; Festuca rupicola; Festuca valesiaca; Holcus lanatus; Lolium perenne; Poa pratensis; Sieglingia decumbens*	*Dorycnium pentaphyllum; Genista tinctoria; Lathyrus pratense; Lathyrus tuberosus; Lathyrus nissolia; Lotus corniculatus; Medicago lupulina; Ononis spinosa; Trifolium alexandrinum; Trifolium pannonicum; Trifolium pratense; Trifolium repens; Vicia cracca; Vicia grandiflora*	*Achillea millefolium; Allium flavum; Allium vineale; Aster linosyris; Campanula rapunculus; Carex vulpine; Carthamus lanatus; Centaurea jacea; Chrysanthemum leucanthemum; Cirsium arvense; Centaurium erythraea; Cerastium fontanum; Cichorium intybus; Clematis integrifolia; Crepis biennis; Eryngium campestre; Falcaria vulgaris; Filipendula vulgaris; Fragaria viridis; Galium mollugo; Galium verum; Filipendula vulgaris; Fragaria viridis; Galium mollugo; Galium verum; Hypericum perforatum; Knautia arvensis; Leonthodon autumnalis; Linaria vulgaris; Luzula arvensis; Mentha arvensis; Origanum vulgare; Ornithogallum umbellatum; Plantago lanceolata; Plantago media; Polygala vulgaris; Potentilla erecta; Prunella laciniata; Prunella vulgaris; Rosa gallica; Rumex acetosella; Rumex crispus; Salvia pratensis; Scabiosa ochroleuca; Senecio jacobea; Stellaria graminea; Tanacetum vulgare; Tragopogon orientalis; Veronica spicata*
2018	*Agrostis tenuis; Agropyron repens; Anthoxanthum odoratum; Botriochloa ischaemum; Bromus hordeaceus; Calamagrostis epigeios; Festuca valesiaca; Holcus lanatus; Poa pratensis; Sieglingia decumbens; Stipa capillata*	*Dorycnium pentaphyllum; Lathyrus pratense; Lathyrus tuberosus; Lathyrus nissolia; Lotus corniculatus; Medicago lupulina; Ononis spinosa; Trifolium pannonicum; Trifolium pratense; Trifolium repens; Vicia grandiflora*	*Achillea millefolium; Aster linosyris; Carduus acanthoides; Carthamus lanatus; Centaurea jacea; Cichorium intybus; Crepis biennis; Cruciata laevipes; Daucus carota; Erygeron anuus; Eryngium campestre; Euphorbia cyparissias; Filipendula vulgaris; Fragaria viridis; Galium mollugo; Galium verum; Geranium pusillum; Hieracium pilosella; Hypericum perforatum; Knautia arvensis; Linaria vulgaris; Marrubium vulgare; Ornithogallum umbellatum; Plantago lanceolata; Plantago media; Polygala vulgaris; Prunella vulgaris; Rumex acetosella; Rumex crispus; Senecio jacobea; Thymus serpyllum; Rosa gallica; Salvia pratensis; Tanacetum vulgare; Xeranthemum inapertum*
2019	*Agrostis tenuis; Agropyron repens; Anthoxanthum odoratum; Botriochloa ischaemum; Bromus hordeaceus; Calamagrostis epigeios; Festuca valesiaca; Poa pratensis; Stipa capillata*	*Dorycnium pentaphyllum; Lotus corniculatus; Medicago lupulina; Trifolium pratense; Trifolium repens; Vicia grandiflora*	*Achillea millefolium; Aster linosyris; Carduus acanthoides; Carthamus lanatus; Centaurea jacea; Cichorium intybus; Crepis biennis; Daucus carota; Erygeron anuus; Euphorbia cyparissias; Filipendula vulgaris; Fragaria viridis; Galium verum; Geranium pusillum; Hieracium pilosella; Linaria vulgaris; Marrubium vulgare; Plantago lanceolata; Plantago media; Polygala vulgaris; Prunella laciniata; Prunella vulgaris; Rosa gallica; Rumex acetosella; Rumex crispus; Senecio jacobea; Thymus serpyllum; Xeranthemum inapertum*
2020	*Agropyron repens; Agrostis tenuis; Alopecurus pratensis; Anthoxanthum odoratum; Botriochloa ischaemum; Brachipodium pinnatum; Briza media; Bromus hordeaceus;* *Calamagrostis epigeios; Cynosurus cristatus; Dactylis glomerata; Festuca arundinacea; Festuca valesiaca; Holcus lanatus; Lolium perenne; Poa pratensis; Sieglingia decumbens; Stipa capillata*	*Dorycnium pentaphyllum; Genista tinctoria; Lathyrus pratense; Lathyrus tuberosus; Lathyrus nissolia; Lotus corniculatus; Medicago lupulina; Ononis spinosa; Trifolium alexandrinum; Trifolium dubium; Trifolium pannonicum; Trifolium pratense; Trifolium repens; Vicia cracca; Vicia grandiflora; Vicia sativa; Vicia tetrasperma*	*Achillea millefolium; Agrimonia eupatoria; Allium flavum; Allium vineale; Aster linosyris; Campanula rapunculus; Carex vulpina; Carthamus lanatus; Centaurea jacea; Chrysanthemum leucanthemum; Cirsium arvense; Centaurium erythraea; Cerastium fontanum; Cichorium intybus; Clematis integrifolia; Crepis biennis; Daucus carota; Eryngium campestre; Euphrasia stricta; Falcaria vulgaris; Filipendula vulgaris; Fragaria viridis; Galium mollugo; Galium verum; Hypericum perforatum; Knautia arvensis; Leonthodon autumnalis; Linaria vulgaris; Luzula arvensis; Marrubium vulgare; Mentha arvensis; Origanum vulgare; Ornithogallum umbellatum; Plantago lanceolata; Plantago media; Polygala vulgaris; Potentilla erecta; Prunella laciniata; Prunella vulgaris; Prunus spinosa; Ranunculus acris; Rosa gallica; Rumex acetosella; Rumex crispus; Salvia pratensis; Scabiosa ochroleuca; Senecio jacobea; Stachys officinalis; Stellaria graminea; Tanacetum vulgare; Thalictrum aquilegifolius; Thymus serpyllum; Tragopogon orientalis; Verbena officinallis; Veronica chamaedrys; Veronica spicata; Xeranthemum inapertum*
2021	*Agropyron repens; Agrostis tenuis; Alopecurus pratensis; Anthoxanthum odoratum; Brachipodium pinnatum; Briza media; Bromus hordeaceus; Calamagrostis epigeios; Cynosurus cristatus; Dactylis glomerata; Festuca valesiaca; Holcus lanatus; Lolium perenne; Poa pratensis; Sieglingia decumbens; Stipa capillata;*	*Dorycnium pentaphyllum; Lotus corniculatus; Medicago lupulina; Trifolium arvense; Trifolium dubium; Trifolium pannonicum; Trifolium pratense; Trifolium repens; Vicia cracca; Vicia grandiflora; Vicia sativa*	*Achillea millefolium; Agrimonia eupatoria; Allium vineale; Carduus acanthoides; Carex vulpine; Carthamus lanatus; Cerastium arvense; Clematis integrifolia; Crepis biennis; Eryngium campestre; Euphorbia cyparyssias; Euphrasia stricta; Falcaria vulgaris; Filipendula vulgaris; Fragaria viridis; Galium verum; Geranium pusillum; Hieracium pilosella; Hypericum perforatum; Knautia arvensis; Linaria vulgaris; Mentha arvensis; Potentilla erecta; Prunus spinosa; Ranunculus acris; Rosa gallica; Rumex acetosella; Salvia pratensis; Stachys officinalis; Stellaria graminea; Thalictrum aquilegifolius; Thymus serpyllum; Veronica hederifolia; Veronica spicata; Xeranthemum inapertum*
